# No Witness, No Clarity: Collateral History and the Puzzle of Psychosis

**DOI:** 10.1192/j.eurpsy.2026.12119

**Published:** 2026-07-31

**Authors:** E. Žderić, A. Čubranić Dominković, G. Rubeša, I. Ljubičić Bistrović

**Affiliations:** Department of Psychiatry, Clinical Hospital Center Rijeka, Rijeka, Croatia

## Abstract

**Introduction:**

In psychiatry, heteroanamnestic data — information gathered from relatives or others familiar with the patient — are often crucial for diagnosis and treatment planning. The absence of such data complicates the evaluation of psychiatric presentations, particularly when substance use and chronic psychosis must be differentiated. This report illustrates the challenges of establishing a diagnosis in a patient with recurrent psychotic episodes, limited collateral information, and possible comorbid alcohol use.

**Objectives:**

To demonstrate the role and limitations of heteroanamnestic information in psychiatric assessment, and to explore the diagnostic dilemmas arising in a patient with first hospital admission in midlife, recurrent psychosis, and unclear substance use

**Methods:**

We present the case of a 49-year-old male, living alone without surviving relatives, with no prior psychiatric treatment. He was admitted to another institution for the first time after destroying property in a psychotic state characterized by hallucinations and grandiose delusions. The only collateral data were from neighbors, who reported he had experienced similar episodes several times annually, though never with aggression. Laboratory findings revealed elevated liver enzymes (AST 114 U/L, ALT 84 U/L, GGT 322 U/L). He was treated for two months, improved clinically, and discharged with a diagnosis of psychosis.

**Results:**

Three months later, the patient was readmitted with similar psychotic symptoms. This time, liver enzymes were normal except for slightly elevated GGT. On examination he appeared flushed, sweating, and mildly ataxic, though he denied alcohol use for six months. Neuroimaging was unremarkable and the Mini-Mental State Examination score was 25/30. With reintroduction of antipsychotic and anxiolytic medication, he rapidly recovered physically and showed partial psychological improvement within one week, though psychotic symptoms persisted in attenuated form.

**Conclusions:**

This case underscores the diagnostic complexity when heteroanamnestic information is limited. Is this a case of chronic psychosis such as schizophrenia, alcohol-related psychosis, or a chronically psychotic patient whose symptoms are exacerbated by alcohol consumption? Without reliable collateral data, clinicians must rely on indirect signs, clinical observation, and cautious longitudinal assessment. The case highlights the indispensable role of collateral information in psychiatric diagnosis and the risks of uncertainty when it is lacking.

**Disclosure of Interest:**

None Declared

